# Congenital Pain-Insensitivity Syndromes in Perioperative Anesthesia and Analgesic Target Identification

**DOI:** 10.3390/ijms27219831

**Published:** 2026-11-02

**Authors:** Alyssa Mckenzie, Dupinder Raman, Neil Shukla, Tiffany Bittar, Nour Fransis, Rachel Dombrower, Nitchanan Theeraphapphong, Sophia M. Mckenzie, Yen Nhi Hoang, Alaa Abd-Elsayed

**Affiliations:** 1 School of Medicine, St. George’s University, West Indies 11739, Grenada; 2 School of Medicine, Uniformed Services University of the Health Sciences, Bethesda, MD 20814, USA; 3 Medical School, Edward Via College of Osteopathic Medicine, Blacksburg, VA 24060, USA; 4 Department of Anesthesiology, University of Wisconsin School of Medicine and Public Health, Madison, WI 53726, USA

**Keywords:** congenital insensitivity to pain, congenital insensitivity to pain with anhidrosis, NTRK1, SCN9A, PRDM12, nociception, voltage-gated sodium channels, autonomic dysfunction, perioperative anesthesia, precision analgesia

## Abstract

Congenital insensitivity to pain (CIP) comprises rare genetic disorders characterized by the absence of perception of noxious stimuli from birth. Despite their shared clinical phenotype, CIP syndromes arise from distinct molecular defects that differentially affect nociceptor development, ion channel function, and autonomic regulation. Pathogenic variants in genes, including NTRK1, SCN9A, and PRDM12, define key mechanisms of human nociception. NTRK1-associated CIP with anhidrosis results from disrupted nerve growth factor signaling, leading to loss of small-diameter sensory fibers, impaired thermoregulation, and autonomic instability. SCN9A- and PRDM12-associated forms affect sodium channel function or nociceptor development; autonomic involvement is generally less prominent but requires assessment in each patient. This narrative review synthesizes the genetic and neurodevelopmental basis of CIP syndromes and examines their implications for perioperative management and the development of analgesics. By correlating genotype-specific mechanisms with clinical phenotypes, CIP syndromes help to identify key components of nociceptive pathways. These rare disorders highlight molecular targets for precision analgesia and demonstrate how genetic characterization may guide perioperative risk assessment and the development of targeted pain therapies.

## 1. Introduction

Rare diseases encompass a wide range of conditions that, together, affect millions of individuals worldwide, despite each occurring infrequently at the population level [1]. Since most are genetic, advancements in genomic tools allow clinicians to identify disease-causing variants, understand how these variants lead to disease, and apply findings towards diagnosis and treatment [2,3]. Investigating phenotypes in individuals with rare diseases can reveal how specific biological pathways function in humans, particularly those that cannot be adequately reproduced in experimental models. Such analysis is particularly useful for examining conditions affecting nociception, where alterations in human genetics can directly affect mechanisms related to pain processing and perioperative management.

Congenital pain-insensitivity syndromes represent a group of rare genetic conditions characterized by the inability to perceive noxious stimuli from birth [4,5]. Though a variety of genes can influence CIP, each mutation can produce a distinct clinical phenotype [4,5]. Autonomic and motor findings vary by genotype and by patient [4,5,6]. Because variations in the syndrome can be distinct, they should not be treated as a single uniform disorder. Rather, each phenotype can be studied individually and serves a role in clinical research based on its specific features and mechanisms.

Congenital insensitivity to pain with anhidrosis is caused by mutations in NTRK1, leading to loss of nociception, impaired thermoregulation, and autonomic instability [7,8]. These features warrant planning for temperature and cardiovascular changes during anesthesia. SCN9A- and PRDM12-associated CIP generally has less prominent autonomic involvement, although associated findings vary [6,9,10]. These conditions help identify molecules required for normal pain signaling and may guide the development of new analgesic therapies.

This narrative review examines congenital pain-insensitivity syndromes as genetically defined conditions that provide complementary insight into nociceptive biology and clinical translation. NTRK1-associated syndromes are analyzed to demonstrate how specific genetic defects affect autonomic and temperature regulation and their implications for perioperative anesthetic management. Examining CIP syndromes with less prominent autonomic involvement provides insight into molecular components of nociceptive signaling and guides the development of new pain treatments relevant to anesthesia and perioperative care. By linking genetic causes of rare diseases to clinical practice, genetic information can be used to anticipate anesthesia-related risks and develop more targeted approaches to precision analgesia.

## 2. Methods

A narrative literature review was conducted to examine congenital insensitivity to pain (CIP), perioperative anesthesia, and analgesic target identification. Contributors performed searches individually over a period of approximately six to eight months before this revision. PubMed, Google Scholar, OpenEvidence-assisted article discovery, and reference lists were used to identify the literature. Search dates, database-specific query histories, and screening decisions were not recorded in a shared log; an exact final date for the original searches could not be reconstructed. The original search terms included “congenital insensitivity to pain,” “CIPA,” “NTRK1,” “SCN9A,” “PRDM12,” “nociception,” “hereditary sensory and autonomic neuropathy,” “anesthesia,” “perioperative management,” and “analgesic targets.” OpenEvidence was used for article discovery, and the evidence synthesized in this review consists of the cited publications.

Selection was based on relevance to congenital pain loss, genotype–phenotype relationships, perioperative outcomes, or analgesic translation. Priority was given to human genetic and functional studies for disease causation, clinical series and case reports for perioperative observations, and experimental studies for proposed biological mechanisms. The reviews supplied context. Publications from the preceding 20 years were emphasized. The literature was organized by topic; no prospectively registered protocol, standardized duplicate screening, formal risk-of-bias assessment, or complete study-selection count was used. The synthesis is therefore selective and does not establish comparative treatment effects or genotype-specific management guidelines.

For this revision, supplementary searches were performed in Europe PMC on 2 October 2026 using three queries: (TITLE_ABS:“congenital insensitivity to pain” OR TITLE_ABS:CIPA) AND (TITLE_ABS:anesthesia OR TITLE_ABS:anaesthesia OR TITLE_ABS:perioperative); TITLE_ABS:“congenital insensitivity to pain” AND (TITLE_ABS:NTRK1 OR TITLE_ABS:NGF OR TITLE_ABS:SCN9A OR TITLE_ABS:SCN10A OR TITLE_ABS:SCN11A OR TITLE_ABS:PRDM12); and (TITLE_ABS:Nav1.7 OR TITLE_ABS:Nav1.8 OR TITLE_ABS:PRDM12) AND (TITLE_ABS:analgesia OR TITLE_ABS:opioid OR TITLE_ABS:nociceptor). Each query was restricted by FIRST_PDATE: [1 January 2006 TO 2 October 2026]. The first 25 records returned for each query were screened for relevance, alongside checks of the existing cited literature. These supplementary searches provided a reproducible revision-stage search and did not reconstruct the original selection process or constitute an exhaustive update.

For the monitoring and regional anesthesia topics added during revision, CIP case reports were considered alongside studies and practice guidance from other perioperative populations. Extrapolations to CIP are identified in the Discussion Section.

## 3. Genetic and Neurodevelopmental Basis of CIP Syndromes

Congenital insensitivity to pain (CIP) syndromes are rare inherited disorders characterized by the absence of perception of noxious stimuli from birth [11,12]. Although affected individuals are unable to perceive pain, other sensory modalities, such as touch, proprioception, and, often, temperature, remain relatively preserved depending on the genetic subtype [11,13]. Advances in human genetics have identified several genes that play essential roles in nociceptive signaling and sensory neuron development [11,14]. Here, CIP denotes inherited loss or marked impairment of nociception beginning at birth or in infancy; related hereditary sensory and autonomic neuropathies are discussed when they clarify this phenotype [4,5,6]. Established causes include NTRK1, NGF, PRDM12, SCN9A, and particular SCN11A variants [6,14]. SCN10A is included as a candidate association and analgesic target: variants have been reported in a congenital sensory/autonomic neuropathy cohort, but pathogenicity and a consistent CIP phenotype require further validation [15]. Detailed discussion focuses on NTRK1 because its autonomic and thermoregulatory manifestations have perioperative evidence; SCN9A because human genetics and functional studies support Nav1.7 as a pain target; and PRDM12 because it illustrates nociceptor development [7,16,17,18,19]. NGF and SCN11A are summarized to define the wider scope; the tables distinguish these established causes from the SCN10A candidate association. These disorders provide insight into the molecular components required for normal human pain perception and for the identification of analgesic targets.

Several forms of CIP arise from mutations affecting voltage-gated sodium channels expressed in peripheral nociceptors [20]. SCN9A encodes the sodium channel Nav1.7, which is highly expressed in dorsal root ganglion neurons and plays a central role in initiating action potentials in response to noxious stimuli [16,21]. Loss of Nav1.7 function can impair peripheral excitability and transmission at nociceptor central terminals; the contribution of each site varies across experimental models [16,22,23,24]. Particular gain-of-function SCN11A variants increase Nav1.9 activity and cause sustained depolarization that impairs action potential generation and synaptic transmission [25]. SCN10A encodes Nav1.8, a pain-related channel whose candidate association with congenital pain loss has less supporting evidence [15,26]. Nav1.8 is particularly enriched in small sensory neurons of the dorsal root ganglia that respond to painful stimuli, and alterations in its function impair repetitive firing of these nociceptors [26,27].

Rather than arising from altered ion channel activity, other variations in CIP syndrome originate from abnormalities in sensory neuron development [11,12]. NTRK1, which encodes the TrkA receptor for nerve growth factor (NGF), is responsible for one of the most recognized forms of the disorder, which is congenital insensitivity to pain with anhidrosis (CIPA) [28]. NGF–TrkA signaling is required during embryonic development for the differentiation and survival of nociceptive neurons [8,18]. Mutations in NTRK1 disrupt this signaling pathway, resulting in a loss or severe reduction in small-diameter sensory fibers, including unmyelinated C fibers and thinly myelinated A-delta fibers that normally transmit pain and temperature signals [7,12]. The absence of these neurons produces the characteristic clinical phenotype of pain insensitivity combined with autonomic dysfunction [11,12].

Developmental forms of CIP, including NTRK1- and PRDM12-associated disease, arise from impaired development or survival of peripheral sensory neurons [8,18,29]. Nociceptive neurons originate from neural crest-derived precursors that migrate to form dorsal root ganglia during embryogenesis [30,31]. Their differentiation into functional pain-sensing neurons depends on transcription factors and neurotrophic signaling, particularly nerve growth factor acting through the TrkA receptor [32,33]. When pathogenic genetic variants disrupt this developmental process, nociceptive neurons may fail to differentiate properly or undergo early cell death, resulting in reduced intraepidermal nerve fiber density. These developmental defects account for congenital pain loss in the affected subtypes; channel-related forms can impair nociception through altered neuronal function [14,16].

**Figure 1:**
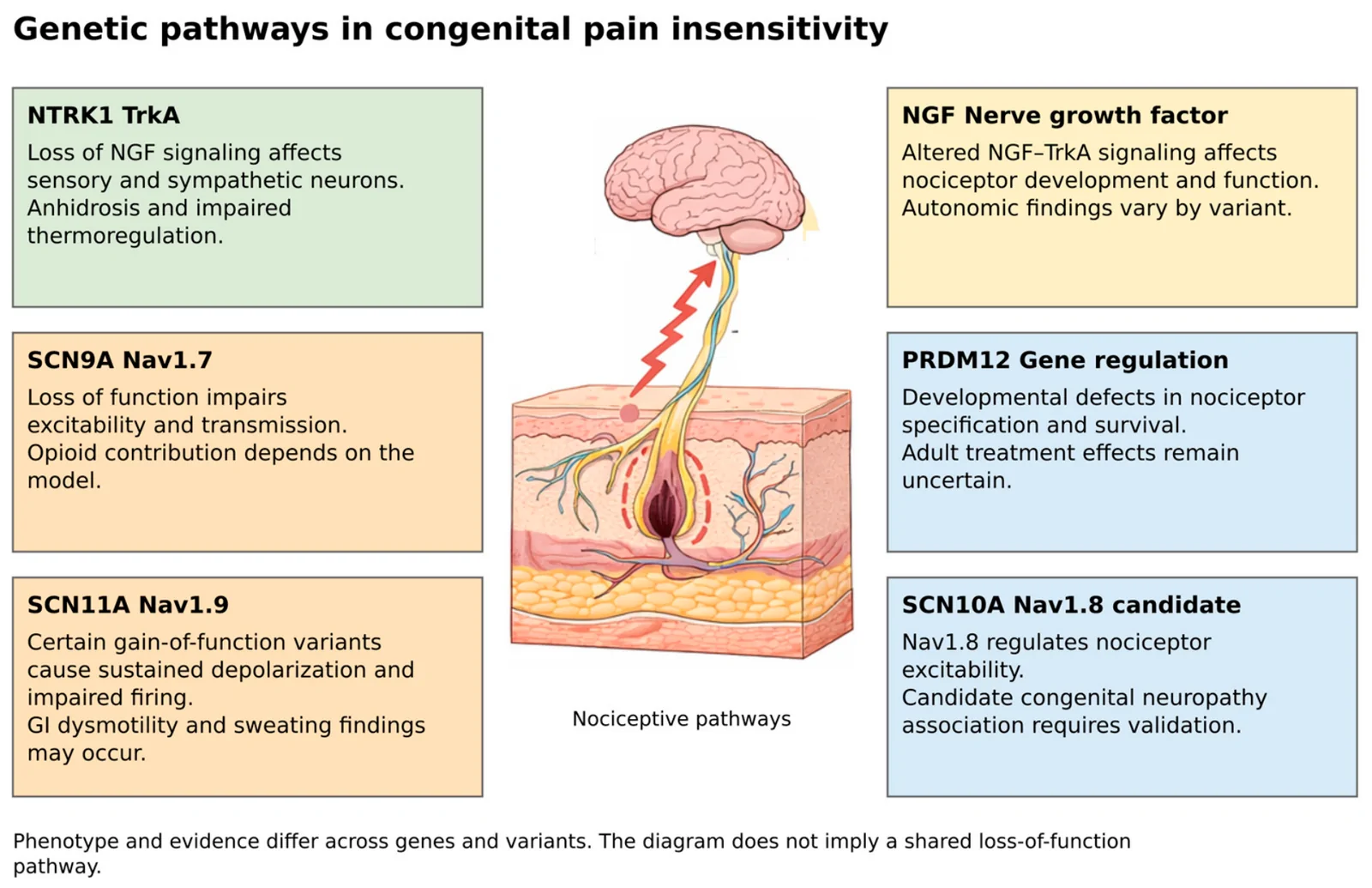
Genetic and neurobiological mechanisms underlying congenital insensitivity to pain syndromes.

Taken together, these genetic disorders demonstrate that pain perception depends on both proper development of nociceptive neurons and intact electrical signaling within those neurons, as illustrated in Figure 1 [5,14]. By linking specific gene defects with defined clinical phenotypes, CIP syndromes help identify key molecular components of nociception and demonstrate how disruption of these pathways leads to loss of pain perception [14]. The major genetic subtypes of congenital insensitivity to pain and their associated clinical and perioperative characteristics are summarized in Table 1.

## 4. Ntrk1-Associated Congenital Insensitivity to Pain with Anhidrosis: Genotype-Specific Perioperative Risk

### 4.1. Ntrk1-Mediated Autonomic Dysfunction and Anesthetic Vulnerability

NTRK1-related CIP with anhidrosis (CIPA) combines sensory and autonomic abnormalities that affect perioperative care [7,17,34]. Disrupted NGF–TrkA signaling causes loss of NGF-dependent sensory neurons and sympathetic postganglionic innervation, including innervation of eccrine sweat glands [8,35,36]. Impaired sweating and cardiovascular regulation warrant assessment of temperature, hemodynamics, hydration, and airway or aspiration risk [17,34,37]. Bradycardia, hypotension, hyperthermia, regurgitation, and suspected aspiration have been described during anesthesia, but their frequency cannot be predicted reliably from genotype alone [17].

### 4.2. Thermoregulation and Temperature Control

Thermoregulatory dysfunction represents an important perioperative consideration in individuals with congenital insensitivity to pain with anhidrosis (CIPA) [34,37]. Loss of sympathetic innervation of eccrine sweat glands impairs sweating and heat dissipation, increasing susceptibility to hyperthermia [8,35,36]. This impaired sweating results from loss of autonomic innervation of eccrine glands associated with NTRK1-related sensory and autonomic neuropathy [8,12]. Consequently, careful intraoperative temperature monitoring is recommended during anesthesia to detect early changes in body temperature and to prevent hyperthermic complications [12,34]. Continuous monitoring methods, such as esophageal temperature probes, have been described in the anesthetic management of pediatric CIPA patients and help maintain normothermia throughout surgical procedures [38]. These reports highlight the importance of vigilant thermoregulation monitoring in reducing perioperative risk in this population [34,39].

### 4.3. Cumulative Perioperative Burden

Patients with congenital insensitivity to pain with anhidrosis (CIPA) often experience a substantial lifetime surgical burden [40,41]. In a retrospective analysis of 35 patients with CIPA followed between 1990 and 2013, a total of 358 procedures were performed, reflecting the frequent need for operative intervention in this population [17]. The high surgical burden is largely attributable to the absence of pain perception, which predisposes individuals to repeated injuries, fractures, delayed clinical presentation, and self-mutilation that may require reconstructive or dental procedures [42,43]. Consequently, these patients are also exposed to repeated episodes of anesthesia [17,44]. Bradycardia and a cardiac arrest were reported in the perioperative series [17]. Repeated procedures provide repeated opportunities for such complications; these data do not establish that prior anesthetic exposure causes a cumulative increase in physiologic vulnerability. Planning should address the patient’s current autonomic and thermoregulatory function and previous anesthetic course [17,34].

### 4.4. Perioperative Complication Management

CIPA warrants planning for cardiovascular instability, impaired thermoregulation, and airway or aspiration complications [17,34]. In the retrospective series of 358 anesthetics in 35 patients, intraoperative bradycardia occurred in 10 procedures (2.8%), and postoperative bradycardia in 11 (3.1%). Hyperthermia above 37.5 °C without preceding fever occurred in three procedures (0.8%); no intraoperative hyperpyrexia above 40 °C was reported. Aspiration was suspected in two patients (0.6% of procedures); a separate regurgitation episode was managed without aspiration. One cardiac arrest required resuscitation [17]. These observations come from one center with repeated procedures in the same patients and should not be treated as population incidence estimates. Management should include temperature surveillance, assessment and treatment of bradycardia or hypotension, access to resuscitation drugs, and airway precautions appropriate to the patient and procedure [17,34]. Temperature control should address both overheating and hypothermia [17,45].

## 5. CIP with Less Prominent Autonomic Involvement and Analgesic Target Identification

### 5.1. Scn9a and Nav1.7 Loss of Function Across the Nociceptive Pathway

SCN9A encodes the voltage-gated sodium channel Nav1.7, which is expressed in sensory and sympathetic neurons and amplifies subthreshold depolarizations [21,46]. Biallelic loss-of-function variants cause congenital pain insensitivity, providing evidence that this channel is required for human nociception [16,47]. Touch and proprioception may be preserved, and widespread autonomic dysfunction is generally less prominent than in CIPA [6,47]. Associated sensory and autonomic findings still require assessment in each patient; anosmia is common, and urinary incontinence has been reported in one family [6,48]. Clinical pain loss cannot be inferred directly from residual current measured in a heterologous expression system [49].

Nav1.7 loss can affect peripheral excitability, axonal signaling, central synaptic transmission, and endogenous opioid signaling. Human patient-derived nociceptors and clinical sensory studies show impaired excitability and loss of functional C-fiber nociceptors [16]. Peripheral nerve recordings in knockout mice also demonstrate changes in heat responsiveness and conduction [24]. MacDonald et al. reported reduced transmitter release from nociceptor central terminals through an opioid-dependent process in a mouse model, with naloxone-reversible analgesia also observed in humans with Nav1.7-null variants [23]. Minett et al. likewise found an endogenous opioid contribution in mice and a human participant [50]. Deng et al., however, demonstrated that loss of Nav1.7 impaired initiation of C-fiber action potentials in mice independently of endogenous opioids [22]. These findings support contributions that depend on species, genetic model, developmental timing, recording site, and assay; they do not establish a single predominant mechanism for every patient with SCN9A-related CIP [16,22,23,24,50]. Protein-interaction studies in mice identified an association of Nav1.7 with Gprin1, an opioid receptor-binding protein [51]. This offers a biological rationale for studying channel–opioid interactions, but it does not establish a perioperative opioid regimen or predict an individual patient’s opioid requirement.

### 5.2. Prdm12 and Nociceptor Development

PRDM12 encodes a transcriptional regulator involved in nociceptor lineage specification and NGF receptor TrkA expression [18,29]. Mouse developmental studies demonstrate loss of the nociceptive lineage after Prdm12 deletion [18,29]. Human biallelic PRDM12 variants cause CIP, and skin biopsies in affected individuals have shown absence of intraepidermal nerve endings [19]. Clinical reports describe variation in residual sensation and associated findings, including reduced tear secretion and occasional anhidrosis [6,10]. PRDM12-associated CIP therefore requires assessment of the phenotype even when autonomic involvement is less prominent than in NTRK1-related CIPA.

The developmental role of PRDM12 supports investigation of gene regulation in nociception. It does not establish that inhibiting PRDM12 in an adult will reproduce congenital pain loss. Landy et al. found that embryonic deletion impaired nociception, whereas deletion in mature mouse nociceptors left most pain responses and injury-induced hypersensitivity intact [52]. Other adult mouse experiments report changes in gene expression and pain-related behavior, indicating that outcomes depend on the deletion model and assay [53]. These findings require distinction between developmental loss of nociceptors and modulation of established neurons.

Targeting PRDM12-related transcriptional or epigenetic pathways for sustained perioperative analgesia remains hypothetical. There is no established PRDM12-directed perioperative treatment, and the cited evidence does not demonstrate reduced opioid use in surgical patients [18,19,52,53]. Translation would require a reversible intervention, demonstration of benefit in adult nociceptors, and evaluation of effects on protective sensation, neuronal survival, and other gene-regulated functions.

### 5.3. Implications for Analgesic Drug Development

Human SCN9A and PRDM12 disorders identify pathways involved in nociception, but complete congenital gene loss differs from partial, reversible pharmacological inhibition [16,19,47,52]. Developmental adaptations, exposure at peripheral and central terminals, target selectivity, and the degree of channel inhibition may affect drug response [16,23,50]. Consequently, inherited painlessness does not by itself establish the efficacy or safety of a drug targeting the same pathway.

Preclinical findings should be attributed to the tested compound and model. McGowan et al. found that the peripherally acting Nav1.7-preferential blocker BZP reduced hyperalgesia and allodynia in rats without causing impairment in the tested motor-coordination assay [54]. ANP-230 blocks Nav1.7, Nav1.8, and Nav1.9, and showed analgesic effects with little motor impairment in animal studies; it is not a selective Nav1.7 inhibitor [55]. The CRMP2–Ubc9 inhibitor compound 194 indirectly modulated Nav1.7 and showed no rewarding or addiction-related effects in the reported rodent tests [56]. These compound-specific observations do not demonstrate that all Nav1.7 inhibitors lack misuse, dependence, or respiratory risks in humans. Reduced CNS exposure may limit some centrally mediated effects, but clinical respiratory safety cannot be inferred solely from peripheral selectivity.

Suzetrigine targets Nav1.8, encoded by SCN10A. The cited suzetrigine reviews therefore concern a separate sodium-channel target and do not support class-wide claims about Nav1.7 inhibitor safety [57,58]. Translation to perioperative practice requires compound-specific human evidence for analgesic efficacy, adverse effects, interactions, and appropriate dosing.

## 6. Implications for Anesthesia and Perioperative Medicine

Congenital pain-insensitivity syndromes challenge the assumption that the absence of pain perception results in a simpler anesthetic course. Autonomic dysfunction is the primary source of perioperative risk in patients with NTRK1-related congenital insensitivity to pain with anhidrosis (CIPA) [17,37]. The anesthetic series and case reports support assessment of airway risk, temperature regulation, and hemodynamics [17,34,37]. These genotype-specific features provide a basis for perioperative risk assessment and intraoperative monitoring strategies.

### 6.1. Perioperative Assessment and Care

**Figure 2:**
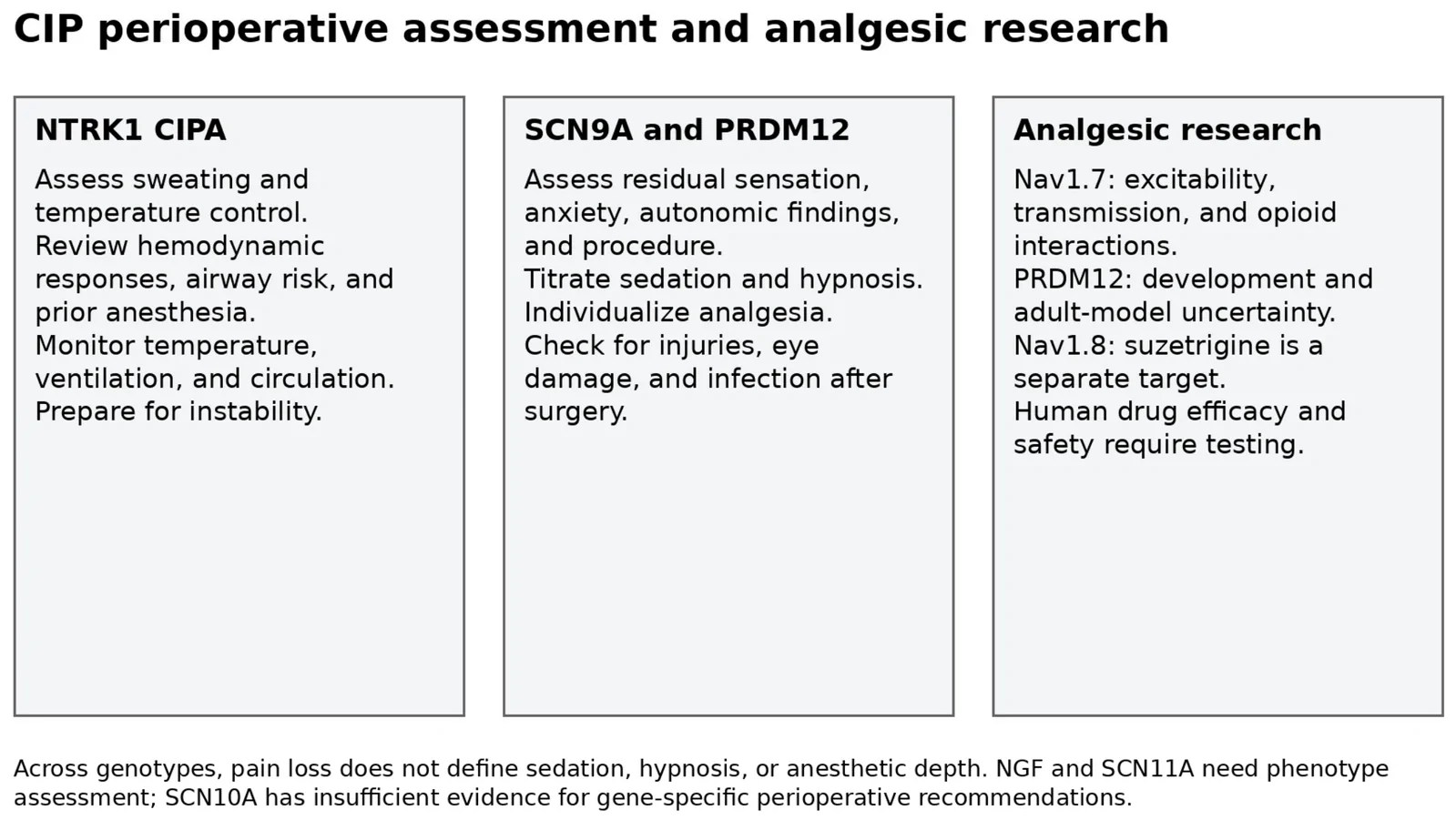
Genotype-specific perioperative risk assessment and analgesic strategies in congenital pain-insensitivity syndromes.

Perioperative assessment should integrate genotype with residual sensory function, sweating, gastrointestinal or urinary symptoms, previous anesthetic responses, anxiety, and the planned procedure (Table 2 and Figure 2). Analgesia treats pain; sedation reduces anxiety or responsiveness; hypnosis provides unconsciousness when required; and anesthetic depth reflects the level needed to meet procedural demands. Congenital pain loss does not determine each of these requirements [37,38,45]. A small series reported volatile-agent requirements within expected ranges, but it did not establish identical requirements for all genotypes or procedures [45]. Sedatives and hypnotics should be titrated to the required level of responsiveness or unconsciousness, with monitoring of ventilation, circulation, and temperature. Opioids should be considered for a defined indication and titrated to effect; they should not be described as a substitute for sedation or hypnosis.

Postoperative analgesia should be guided by residual or newly reported pain, discomfort, procedural factors, and clinical findings. Case evidence describes postoperative autonomic responses treated with analgesics even in a patient with congenital pain insensitivity [59]. Absence of pain can also delay recognition of fractures, pressure injuries, corneal damage, or infection, so examination and physiologic surveillance remain important [6,40,59]. Evidence for SCN9A- and PRDM12-specific perioperative drug requirements is limited; the table provides considerations for assessment rather than validated management protocols.

SCN9A studies support peripheral and central contributions to pain loss, with endogenous opioid involvement in some models [16,22,23,47,50]. PRDM12 studies identify developmental pathways whose relevance to adult treatment requires further testing [18,19,52,53]. These observations can guide research into analgesic targets while clinical planning remains based on the patient’s phenotype and procedure.

### 6.2. Digital Monitoring of Anesthesia

When monitoring the depth of anesthesia, the Bispectral Index (BIS), EEG entropy (state and response entropy), and auditory evoked potential indices can provide adjunct information about the hypnotic component of anesthesia. BIS has been used in reports of anesthesia for CIPA [34,37,38]. Pain insensitivity does not establish unconsciousness or exclude awareness. Processed indices should therefore be interpreted with the EEG or evoked response, anesthetic delivery, and the clinical setting. Drug effects and electromyographic activity can affect their readings; a study in patients without CIP demonstrated different responses of BIS, entropy, and an auditory evoked potential index to ketamine and rocuronium [60]. CIP-specific target values and outcome benefits are not established by these reports. These devices supplement routine physiological monitoring and do not directly measure pain or neuromuscular recovery.

In nociception and antinociception monitoring, the Surgical Pleth Index (SPI) combines pulse wave amplitude and heartbeat interval, whereas the Analgesia Nociception Index (ANI) uses heart-rate variability to estimate changes in autonomic activity [61]. The Nociception Level (NOL) index combines several physiological signals, including heart-rate variability, pulse wave amplitude, and skin conductance [62]. A published study protocol also describes peripheral skin temperature among the measurements obtained by the NOL finger probe [63]. Sensor measurements and algorithm inputs should be distinguished for the device version in use. Peripheral skin temperature does not replace core temperature monitoring in CIPA. These indices estimate autonomic responses associated with nociception and analgesic administration; their readings do not establish the patient’s experience of pain. Studies in patients without CIP support investigation of these approaches, but do not establish thresholds or treatment benefits for CIP [61,62].

NTRK1-related CIPA requires particular caution when interpreting these indices. Anhidrosis and sympathetic dysfunction may alter skin conductance, cardiovascular responses, and temperature regulation [5,8]. Consequently, the physiological features highlighted for NOL warrant genotype-specific validation before its readings are used to guide analgesic dosing. Vasoactive drugs, sedatives, peripheral perfusion, and temperature should be recorded as potential influences on autonomic signals. A low index should not be taken as proof that all procedural stimuli are adequately managed, and an elevated index should prompt assessment of its cause before analgesic escalation. Future studies should compare baseline and stimulus-related readings across genotypes, residual sensation, and autonomic phenotypes, and assess whether index-guided care changes drug exposure or perioperative outcomes.

Neuromuscular transmission monitoring. When neuromuscular blocking drugs are used, quantitative monitoring should guide dosing, reversal, and recovery assessment. The 2023 American Society of Anesthesiologists guidelines recommend monitoring at the adductor pollicis and confirming a train-of-four ratio of at least 0.9 before extubation [64]. Reversal should be selected according to the drug and measured depth of blockade. These recommendations are extrapolated from general perioperative practice. Congenital pain loss does not establish recovery of muscle strength or airway protection. Neuromuscular monitoring assesses motor blockade separately from hypnotic depth and nociceptive responses; clinical signs alone should not substitute for quantitative recovery assessment [64].

### 6.3. Regional Anesthesia Techniques

Regional anesthesia may be considered according to residual sensation, tactile discomfort, positioning needs, motor blockade requirements, and the planned procedure. A report of an NTRK1-related CIPA patient undergoing hip surgery described ultrasound-guided femoral and lateral femoral cutaneous nerve blocks with dexmedetomidine sedation [65]. Spinal anesthesia has also been reported in CIPA [66]. These reports establish that regional techniques have been used, while their effects on outcomes and comparative advantages remain uncertain. Regional anesthesia does not remove the need to assess anxiety, consciousness, autonomic function, and temperature. The indication and expected benefit should be explained for the individual patient.

Before a regional technique, baseline sensory and motor findings should be documented and the usual procedural contraindications assessed. Existing neuropathy warrants discussion of neurological risk and a plan for assessment after the procedure [67]. Ultrasound can assist needle placement for peripheral blocks, but does not eliminate injury risk. Pain reporting may be unavailable as a warning during injection or as a measure of block success; assessment should therefore use the expected motor findings, procedural conditions, and monitoring appropriate to the technique. Neuraxial sympathetic blockade may aggravate hypotension in patients with autonomic dysfunction, so hemodynamic and temperature monitoring remain necessary. Surveillance for local anesthetic toxicity and protection of an insensate or temporarily weak limb should continue after the procedure. These considerations require patient-specific judgment because CIP-specific comparative evidence is limited [65,66,67].

## 7. Limitations

This narrative review has several limitations related to the available literature and the scope of the review design. Since congenital pain-insensitivity syndromes are rare, the available evidence is largely derived from case reports, small cohort studies, and retrospective analyses [11,68]. Additionally, phenotypic and genotypic variation exists across congenital pain-insensitivity syndromes [69,70]. Even within single-gene mutations such as NTRK1, clinical expression may vary widely between patients, complicating the establishment of standardized perioperative protocols [5,8]. Although genetic studies have identified the molecular causes of congenital pain-insensitivity syndromes, the full clinical implications of many mutations during anesthesia and surgery remain unclear. Existing anesthesia outcome data in patients with congenital pain-insensitivity syndromes are largely limited to small retrospective case series, and long-term cumulative anesthetic exposure outcomes remain poorly characterized [45]. The original searches were performed individually without a dated shared log or prospectively specified selection protocol. The dated supplementary searches were limited to the first 25 records returned by each query and checks of the cited literature. Selection and ranking bias, incomplete coverage, and the absence of formal risk-of-bias assessment limit reproducibility and preclude claims of an exhaustive synthesis. Experimental analgesia and safety findings may not generalize to humans, and genotype alone does not define perioperative drug requirements. Evidence for digital monitoring and regional anesthesia in CIP is also limited: the cited CIP literature consists of case reports, while monitor comparisons and neuromuscular recommendations largely come from other populations. Genotype-specific validation and comparative outcome studies are needed before benefits can be assumed.

## 8. Future Directions

Advancements in genetic testing and analysis are making it easier to classify rare genetic diseases, thereby providing more opportunities to integrate molecular data into perioperative care. Once the precise genetic cause of a given congenital pain syndrome is identified and accurately interpreted, this information can be used to anticipate clinical risks and guide the development of new treatments.

For congenital pain syndromes that affect the autonomic nervous system, such as those caused by NTRK1 mutations, future research should aim to develop standardized guidelines for genetic data in perioperative evaluation. If genetic diagnoses are determined before surgical intervention, clinicians may be better able to predict and prepare for complications such as unstable blood pressure, abnormal heart rate responses, or impaired temperature control during anesthesia. Systematically studying the responses of different genetic variants in patients during surgery can help researchers to identify genotype-specific risk patterns and determine which monitoring strategies are most appropriate.

For congenital pain syndromes with less prominent autonomic involvement, additional investigation of the underlying genes and their role in nociceptive signaling may help to identify specific molecular targets for more precisely targeted analgesic therapies. The integration of mutated genes with transcriptional genes and their effects on nociceptive function can help identify the principal molecular targets for developing new pain treatments.

Prospective studies should evaluate BIS, EEG entropy, auditory evoked potentials, SPI, ANI, and NOL in patients with defined CIP genotypes. Baseline autonomic function, residual sensation, temperature, anesthetic delivery, and drugs affecting autonomic signals should be recorded alongside monitor readings. Outcomes should include awareness, hemodynamic instability, temperature disturbances, drug exposure, and recovery. When neuromuscular blocking drugs or regional techniques are used, quantitative recovery measurements and neurological findings should also be recorded. Such studies would test whether these approaches improve care and identify which signals remain interpretable in NTRK1-related anhidrosis.

Collaboration among experts in genetics, anesthesia, and clinical research can transform discoveries in rare disease genetics into practical strategies for patient care. As genetic testing becomes more prevalent in everyday medical practice, these rare congenital pain syndromes can serve as examples of how genetics can be used to improve surgical planning and develop more individualized pain treatments.

## 9. Conclusions

Congenital pain-insensitivity syndromes encompass a combination of rare disease genetics, nociceptive neurobiology, and perioperative clinical medicine. Although these disorders are rare, they provide insight into the molecular mechanisms of pain perception and autonomic regulation. From a perioperative perspective, an improved understanding of genotype-specific risk patterns may enhance clinicians’ awareness of complications related to autonomic instability, thermoregulation, and other perioperative risks. SCN9A- and PRDM12-associated CIP identify pathways for analgesic research. Their translation to adult treatment requires compound-specific efficacy and safety evidence, and perioperative care should be tailored to phenotype and procedural requirements. Continued study of congenital pain-insensitivity syndromes may improve understanding of nociceptive mechanisms and support more individualized approaches to perioperative care.

## Figures and Tables

**Table 1:** Genetic subtypes of congenital pain insensitivity and perioperative implications. * SCN10A denotes a candidate association; established causes are distinguished [6,14,15,32].

Gene	Encoded Protein	Pathophysiologic Mechanism	Key Clinical Features	Autonomic Involvement	Perioperative Considerations
NTRK1	TrkA receptor	Impaired NGF signaling → loss of nociceptive neurons [8]	Pain insensitivity, anhidrosis, thermoregulation defects [6,7]	Yes [6,7]	Temperature monitoring, hemodynamic instability, aspiration risk [17]
SCN9A	Nav1.7 sodium channel	Impaired excitability and central transmission; model-dependent opioid contribution [16,22,23]	Complete absence of pain perception [6]	Usually less prominent; assess phenotype [6]	Assess sensation, anxiety, and procedural requirements [6]
PRDM12	Transcription factor	Failure of nociceptor lineage specification [18,19,29]	Reduced intraepidermal nerve fiber density [19]	Usually less prominent; variable sweating/tear findings [6,10]	Assess sensory function, corneal protection, and procedural requirements [6,10]
SCN10A *	Nav1.8 sodium channel	Nav1.8 affects excitability; congenital disease association requires validation [15,26,27]	Candidate sensory/autonomic neuropathy association [15]	Uncertain; assess reported phenotype [15]	Insufficient evidence for gene-specific recommendations [15]
SCN11A	Nav1.9 sodium channel	Certain gain-of-function variants cause sustained depolarization and impaired firing [25]	Pain insensitivity; intestinal dysmotility and hyperhidrosis can occur [6,25]	Variable; gastrointestinal and sweating features reported [6,25]	Assess gastrointestinal and autonomic findings; limited anesthesia evidence [6]
NGF	Nerve growth factor	Impaired NGF–TrkA signaling; variant-dependent sensory neuron effects [32,33]	Congenital pain loss; sensory and other findings vary [6,32]	Variable by variant [6]	Assess sweating and sensory function; limited anesthesia evidence [6]

**Table 2:** Perioperative management and monitoring in congenital pain-insensitivity syndromes. * SCN10A denotes a candidate association. These considerations are extrapolated from phenotype reports and limited perioperative series; they are not genotype-validated protocols [6,10,15,17,37,38,45,48,59]. Citations within each row identify phenotype evidence and the sources used for perioperative extrapolation; general anesthesia evidence [45] does not establish genotype-specific requirements.

Genetic Subtype	Risks and Evidence Limits	Monitoring and Assessment	Sedation Hypnosis and Anesthetic Depth	Analgesia and Postoperative Assessment
NTRK1	Impaired thermoregulation; bradycardia/hypotension; airway or aspiration complications [17,34,37]	Core temperature, ECG, BP, oxygenation and ventilation; assess aspiration risk [17,34,37]	Titrate sedation/hypnosis to anxiety and procedure; anticipate circulatory instability [37,38,45]	Assess residual sensation and discomfort; use analgesics for a defined indication; monitor for injury/infection [6,37]
SCN9A	Unrecognized injury; autonomic findings require assessment; genotype-specific anesthesia evidence limited [6,48]	Routine anesthesia monitoring; assess residual sensation, injuries, and urinary/autonomic history [6]	Titrate sedation/hypnosis to anxiety and procedure; assess required depth [45]	Requirements may be reduced; do not assume zero need; assess symptoms and postoperative complications [6,45]
PRDM12	Injury/corneal damage; sweating or tear abnormalities reported; perioperative evidence limited [6,10]	Routine anesthesia monitoring; assess sensation, sweating, tears, and injuries [6,10]	Titrate sedation/hypnosis to anxiety and procedure; protect eyes and pressure points [6,45]	Assess residual sensation/discomfort; individualize treatment and surveillance for injury/infection [6,10]
SCN10A *	Disease association and perioperative risks insufficiently established [15]	Routine anesthesia monitoring; assess the patient’s sensory/autonomic phenotype [15]	No established gene-specific regimen; titrate to the procedure and patient [15,45]	Base treatment on residual pain and clinical findings; evidence does not support a genotype-only prescription [15]
SCN11A	Variant-dependent phenotype; intestinal dysmotility and hyperhidrosis may occur; anesthesia data limited [6,25]	Routine anesthesia monitoring; assess GI function, hydration, temperature and autonomic findings [6,25]	Titrate sedation/hypnosis to anxiety and procedure; assess airway/aspiration risk [6,45]	Individualize to residual pain and clinical findings; assess injury and gastrointestinal complications [6,25]

## Data Availability

No new data were created or analyzed in this study. Data sharing is not applicable to this article.
